# Seasonal Variations of Sediment Fungal Community of a Shallow Lake in North China

**DOI:** 10.3390/microorganisms12112127

**Published:** 2024-10-24

**Authors:** Yujun Yi, Senlu Yin

**Affiliations:** 1 State Key Laboratory of Water Environmental Simulation, School of Environment, Beijing Normal University, Beijing 100875, China; 2 Ministry of Education Key Laboratory of Water and Sediment Science, School of Environment, Beijing Normal University, Beijing 100875, China

**Keywords:** lake sediment, fungi community, Baiyangdian Lake, high-throughput sequencing technique

## Abstract

Fungi play important roles in the process of material cycling and energy transfers in aquatic ecosystems. Yet, little is known about the fungal community in lake sediment. In this study, sediment samples from five habitat types in Baiyangdian Lake (BYD Lake) were collected across three seasons. High-throughput sequencing techniques were used to determine the compositions of fungal communities. Fungi are highly diverse in the sediment of BYD Lake, although some important fungi have not been accurately identified. The fungal diversity was highest in winter and lowest in summer, while there was no significant difference in species richness among sampling sites. The compositions of fungal community differed among seasons and habitats. Physicochemical properties of sediments were measured and the influence of the environmental factors on fungal communities were analyzed. Temperature, P, N, and heavy metals explained 48.98% of the variations of fungal communities across three seasons. Human activities have affected the species and biomass of fungi to some extent. Temperature is the most influential factor and negatively correlated to fungal diversity. Nutrients in different forms have different effects on shaping the fungal community. The effect of heavy metals is relatively low.

## 1. Introduction

As an important component of global aquatic ecosystems, freshwater lakes provide habitats for animals and plants and support global material cycling [1,2]. However, freshwater lakes, especially shallow lakes, are susceptible to climate change and human disturbances, resulting in water scarcity, area loss, overexploitation, pollution, and ecological degradation [3,4]. Sediment is an essential component of shallow lake ecosystems. It is not only a reservoir of various substances, but also an important interface for water–soil physical, chemical, and biological reactions [5]. The highly heterogeneous spatial structure and complex chemical composition of organic and inorganic nutrients in the sediment provide diverse microhabitats for biological groups [6] so that sediment have high biomass and biodiversity. Fungi constitute a substantial proportion of microorganisms in the sediment and play a driving role in the process of material cycling and energy transfer [7,8]. However, owing to methodological limitations, the systematic analysis of fungal diversity and the ecological roles of fungi in shallow lake ecosystems had not progressed for years [9].

The application and popularization of high-throughput sequencing technology and related methods provided powerful tools for studying microbiotic diversity [10]. A large number of fungal species that were difficult to observe by morphology or culture methods have been discovered, which greatly enriches the research on microbial diversity in ecological environments. The development of bioinformatics also facilitates the studies of the classification, functions, and interrelationships of microorganisms and microbial communities [11]. However, little is known about the compositions of fungal communities and their ecological roles [12], especially in lake sediment. Moreover, there are also gaps in knowledge regarding the composition and dynamics of fungal communities in response to the physical and chemical properties of sediments and pollutant concentrations, which are affected by human activities. Therefore, the study of fungal communities in sediments is helpful to reveal the mechanisms by which they are involved in ecological lake processes, as well as to exploring the overall impact of human activities on shallow lake ecosystems.

## 2. Materials and Methods

### 2.1. Study Area and Sampling

Baiyangdian Lake (BYD Lake) is the largest freshwater lake in the North China Plain and has important ecological functions such as climate regulation, water storage, fishery production, biodiversity maintenance, etc. [13]. This macrophytic lake, which consists of over 100 small and shallow lakes linked to each other by thousands of ditches, with a total area of 366 km^2^, is located 100 km southeast of Beijing and 40 km east of the city of Baoding (38°43′ N~39°02′ N, 115°45′ E~116°07′ E). According to the administrative divisions, 39 villages are located within or around the lake. The lake suffered from eutrophication during the 2000s as agricultural, aqua cultural, and residential sewages were directly discharged into the lake [14].

**Figure 1:**
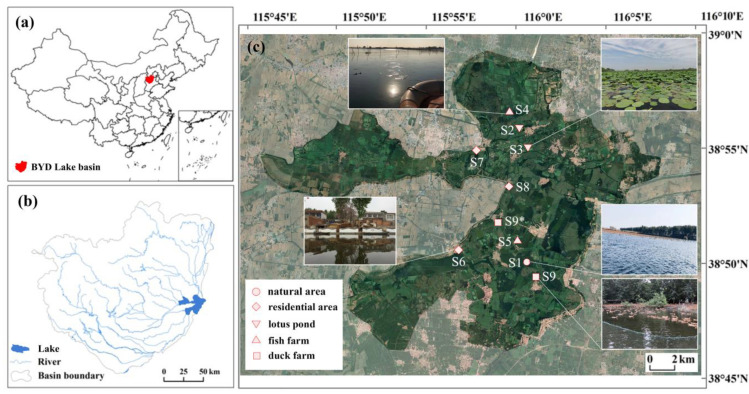
Sampling sites of BYD Lake: (**a**) location of BYD Lake basin in China, (**b**) location of BYD Lake in the basin, and (**c**) sampling sites and views of different habitats.

Nine sampling sites with different disturbance levels of human activities were selected in this study (Figure 1). The sites included most typical habitats (natural areas, residential areas, lotus ponds, fish farms, duck farms) of BYD Lake. Site 1 (S1), located in the Baiyangdian National Aquatic Germplasm Reserve; S2 and S3, located in lotus ponds around villages; S4, located in a fish farm with area of 10 hm^2^ and S5, in another one over 100 hm^2^; S6, S7, and S8, located within or around villages with 10,000, 1000, and 4000 inhabitants, respectively; S9, located in a duck farm which was removed in 2018 and was replaced by another duck farm S9*. Sediment samples were collected in December 2017, March 2018, and July 2018, which represented winter, spring, and summer, respectively, with three parallel samples at each site. Sediments were collected using a Petersen mud grabber, and at least two tanks of mud from the top 0–3 cm of sediment were collected for each sample. Muds were mixed, cooled, and quickly sent to the lab to be stored at −20 °C.

### 2.2. Determination of Physicochemical Properties and Heavy Metals

The temperature of sediment was measured at the same time as sampling occurred. The sediment pH level was measured in a slurry of sediment and water (1:2.5) using a pH meter (ST3100/F, Parsippany, NJ, USA). The contents of carbon (C) and nitrogen (N) were detected using an elemental analyzer (VARIO EL, Langenselbold, Germany). Before detection, the inorganic carbon in the sediment was removed by adding 5 mL of 1 mol/L hydrochloric acid (HCl) to a sample of 2 g of sediment, and then the sample, as well as 2 g of the raw sample, was oven-dried at 105 °C [15]. Dried samples were weighed to obtain the ratio of raw sediment to acidified sediment, and then the acidified sample was ground and passed through a 100-mesh sieve. The contents of total nitrogen (TN) and total organic carbon (TOC) of each sample were recalculated by multiplying the ratio of raw sediment to acidified sediment. Then, 100 mL of a 2 mol/L KCl solution was added to 10 g of sediment, and the mixture was shaken for 30 min to measure the concentrations of nitrate (NO_3_^−^) by comparing the absorbance [16]. Ammonium nitrogen (NH_4_^+^) content was determined by continuous air-segmented flow analysis using a Skalar SAN Plus segmented flow analyzer (Skalar Inc., Breda, The Netherlands). Inductively coupled plasma atomic emission spectrometry (SPECTRO ARCOS EOP, Wilmington, MA, USA) was used to analyze the contents of total phosphorus (TP) and heavy metals (arsenic (As), cadmium (Cd), chromium (Cr), copper (Cu), lead (Pb), zinc (Zn), cobalt (Co), manganese (Mn), nickel (Ni), and iron (Fe) of each sample, which were digested with nitric acid-hydrofluoric acid-hydrochloric acid (HNO_3_-HF-HCl) in an automatic digestion instrument (POLYTECH ST-60, Beijing, China). The contents of different phosphorus forms included the loosely sorbed P (NH_4_Cl-P), the reductant-soluble P (BD-P), the aluminum oxides-associated P (NaOH-P), the calcium-bound P (HCl-P), and the organic insoluble P (Res-P), which is most difficult to release [17], derived from the summary of reference [18].

### 2.3. DNA Extraction, PCR Amplification and Sequencing Environmental Is Acidic

Sediment DNA was extracted from 0.4 g samples using a Fast DNA Spin Kit (Mobio, San Mateo, CA, USA) according to the instructions. The primers SSU 1196R and SSU 0817F [19] were used to amplify the 18S rRNA gene of the fungi. The PCR products were purified by the AxyPrep DNA Gel Extraction Kit (Axygen Biosciences, Union City, CA, USA) and quantified by the QuantiFluorTM-ST (QuantiFluor, Madison, WI, USA) instrument and then sequenced on the Illumina MiSeq platform (Illumina, San Diego, CA, USA). MOTHUR [20] was used to filter, stitch, and control the original sequencing data. Sequences with 97% similarity were clustered into operational taxonomic units (OTUs) using UCLUST [21]. The OTUs were compared with the Unite 18S rRNA database [22] to obtain the classifications of the OTUs. The China Biological Species List (2020 version) [23] and Species 2000 (2019 version) [24] were referred to for classifying uncertain species.

### 2.4. Statistical Analyses

Statistical analyses were implemented in R (version 3.6.2, https://www.r-project.org/ (accessed on 10 April 2020)). The two-way analysis of variance test (two-way ANOVA) was used to determine the differences in the contents of heavy metals, physicochemical properties, and microbial alpha diversity indices among sampling sites and seasons with the car package (version 3.1-3). The α diversity indexes (Sob (species observed), Chao1, Simpson, and Shannon Index) were calculated with the picante package (version 1.8.2). Principal co-ordinates analysis (PCoA) and analysis of similarities (ANOSIM) were used to analyze the β diversity of different sites and seasons with the vegan package (version 2.5-7). Redundancy analysis (RDA) was used to investigate the influence of the environmental factors on fungal communities with the vegan package, and the multicollinearities of different parameters were modified by checking the variance inflation factors (VIFs). Pearson relationship was used to analyze the relationships of the environmental factors with the Hmisc package (version 4.5-0). Figures were drawn with Origin (version 2017, OriginLab, Northampton, MA, USA) and R.

## 3. Results

### 3.1. Sediment Physical and Chemical Properties

The physical and chemical properties of the sediments are shown in Table 1. The concentrations of total phosphorus (TP), HCl-P, As, Cd, Co, Zn, and Mn showed no significant difference among seasons, while others varied significantly. The values of environmental factors of each site are shown in Figure S1. The results also indicate significant differences among sites. For example, S2 and S3 had higher concentrations of total nitrogen (TN), total organic carbon (TOC), NH_4_^+^, Cu, and Ni. S9* had highest concentrations of TP, Res-P, NaOH-P, and HCl-P, while it had lower NH_4_^+^, As, Pb, Co, and Mn. S6, S7, and S8 had higher NH_4_Cl-P and Fe.

The results of Pearson relationships are shown in Table S1. The concentrations of some factors were highly correlated, for example, TP and HCl-P (r = 0.98, *p* < 0.01), TN and TOC (r = 0.96, *p* < 0.01), Cu and Ni (r = 0.83, *p* < 0.01), and Co and Fe (r = 0.88, *p* < 0.01), indicating that it is difficult to distinguish these factors that affect fungal communities.

### 3.2. Compositions of Fungal Community in Sediments

**Figure 2:**
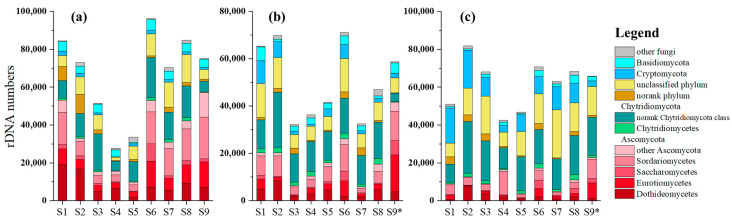
Sequence numbers of fungi in sediment of BYD Lake: (**a**) winter, (**b**) spring, (**c**) summer.

There were 1,110,680, 851,215, and 989,346 sequences obtained in winter, spring, and summer, respectively. These sequences were clustered into 772, 1031, and 1118 OTUs, respectively, and the OTUs were identified into 537 species in total. We filtered 111, 99, and 89 species as fungi from these species, accounting for 53.8%, 53.1%, and 55.4% of sequences in winter, spring, and summer, respectively. The sequence numbers of fungi in different seasons and different sites are shown in Figure 2. Sequence numbers and community compositions variated among seasons and sites. The fungi mainly belonged to Ascomycota and Chytridiomycota, accounting for 34.4% (7.7~76.3%) and 18.9% (4.5~34.7%) of all fungi sequences, respectively. Dothideomycetes, Eurotiomycetes, and Sordariomycetes were the most abundant classes of Ascomycota, while most Chytridiomycota belonged to a norank class. Basidiomycota, Blastocladiomycota, and Cryptomycota accounted for 3.5% (0.7~6.8%), 1.1% (0.04~3.0%), and 6.2% (0.4~23.9%), respectively. Little Glomeromycota, Neocallimastigomycota, or zygosporic fungi were obtained, adding up to less than 1% of the fungi sequences. In addition, 2.6% (0.1~12.0%) and 12.7% (2.9~30.9%) sequences belonged to a norank phylum and an unclassified phylum, respectively.

**Figure 3:**
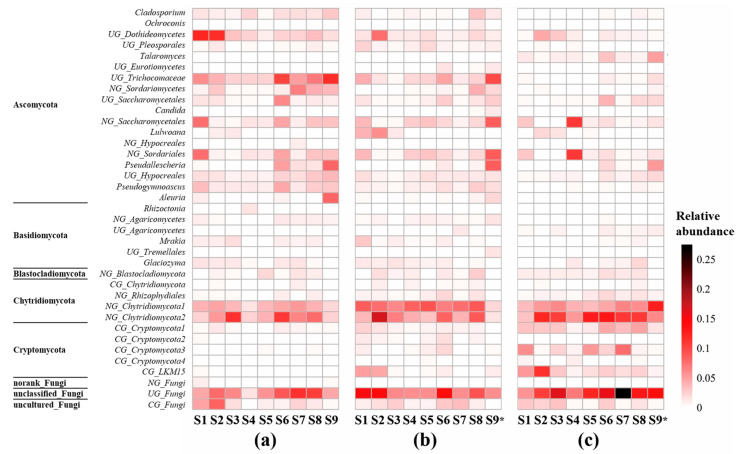
Relative abundance of fungi genera in sediment of BYD Lake: (**a**) winter, (**b**) spring, (**c**) summer. Note: NG—norank genus, UG—unclassified genus, CG—uncultured genus.

The patterns of dominant genera, which had relative abundances over 1% in at least one site, are shown in Figure 3. There were 37 dominant genera in total and 28, 29, and 25 in winter, spring, and summer, respectively, covering 93.5~99.4% sequences. Dominant genera across all sites in winter, spring, and summer were 9, 4, and 3, respectively. *Glaciozyma* (Basidiomycota), a norank Blastocladiomycota genus (*NG_Blastocladiomycota*), and an uncultured Cryptomycota genus (*CG_Cryptomycota3*) dominated all sites and all seasons. The percentages of these three genera accounted for 16.40% (5.42~24.23%), 22.77% (9.18~38.85%), and 28.85% (12.43~44.72%) in winter, spring, and summer, respectively.

### 3.3. Fungal Diversity in Sediment

**Figure 4:**
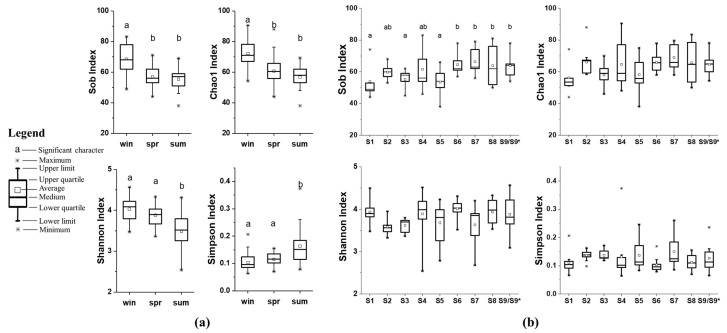
The α diversity indexes of fungi: (**a**) different seasons, (**b**) different sites. Note: win: winter, spr: spring, sum: summer. Characters (a, b) indicate significant difference.

The α diversity indexes are shown in Figure 4. The ranges of the Sob, Chao1, Shannon, and Simpson indices for all samples were 38~83, 38.0~90.5, 2.54~4.57, and 0.06~0.37, respectively. The Sob index and Chao1 index were higher in winter, indicating more species detected. A significantly lower Shannon index and a higher Simpson index in summer indicated lower fungal diversity in this season. The fungal diversity was highest in winter and lowest in summer, and in spring the biodiversity was in between these. The differences of indexes among sites were not as significant. The Sob indexes of S1, S3, and S5 were significantly lower than S6, S7, S8, and S9 (S9*), while the Chao1, Shannon, and Simpson indexes among sites showed no significant differences.

**Figure 5:**
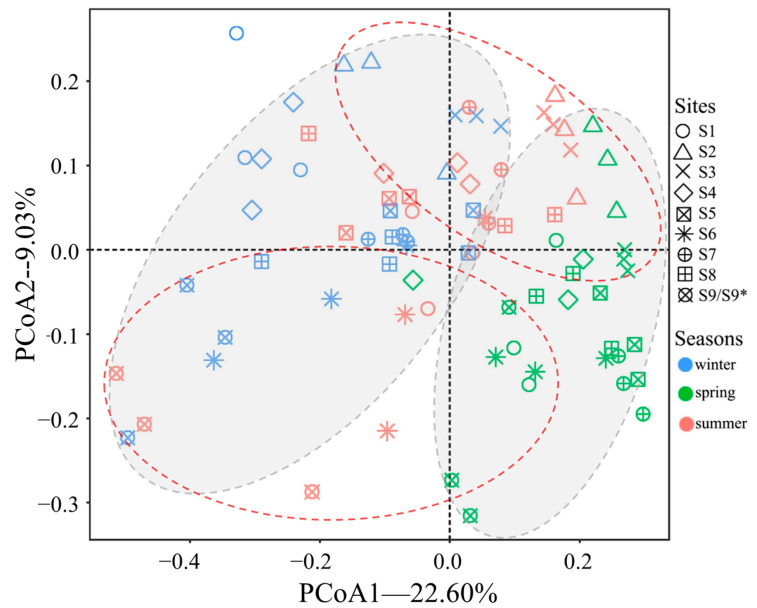
PCoA results of the fungal communities. The 1st (PCoA1) and 2nd (PCoA2) dimension interpreted 22.6% and 9.03% of the differences of samples, respectively. Gray oval zones cluster most samples from the same season. Dashed red oval zones cluster most samples from the same habitat type.

The results of PCoA are shown in Figure 5. For seasonal analysis, winter and summer samples (in gray oval zones) were generally clustered into two groups, which were distributed along the two sides of the first axis, and the spring samples were scattered among them. For sampling sites, most samples mixed, but lotus ponds (S2 and S3) and duck farms (S9 and S9*) were clustered into two groups (dashed red oval zones). ANOSIM results (Figure S2) showed significant variations of fungal communities among both sites and seasons (*p* = 0.001), but when all species in the same season were composed and contrasted, the variations among seasons were low (R = 0.085).

### 3.4. RDA of Environmental Factors and the Fungal Community

**Figure 6:**
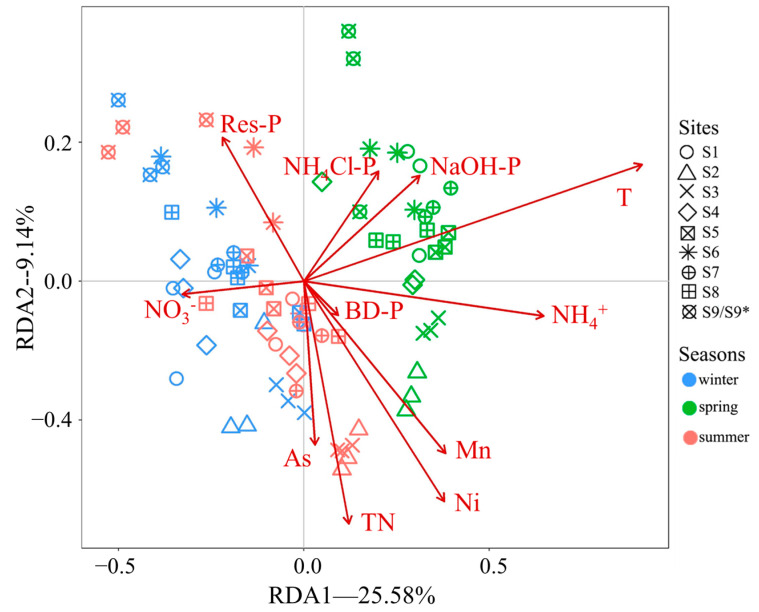
RDA analysis results of the influence of environmental factors on the fungal communities in BYD Lake sediments. The percentages of *X*-axis (RDA1) and *Y*-axis (RDA2) explained 22.58% and 9.14% of the differences in the original data, respectively. The arrows projected on the axis represent the correlations between environmental factors and the distribution of samples. The angle between the arrow line and the axis represents the degree of correlation between the factor and the axis (RDA1 and RDA2). The length of the arrow line indicates the degree of correlation between factors and the distribution of samples.

The influences of environmental factors on the fungal communities of the BYD Lake sediments are shown in Figure 6. Interpretation rates of significant environmental factors are shown in Table 2. Parameters had significant effects explaining 48.98% of the variations across three seasons, and the first and second axes accounted for 34.72%. The influence of temperature was the greatest. Phosphorus, including forms of Res-P, NaOH-P, BD-P, and NH_4_Cl-P, had the second greatest influence, accounting for 11.67% of the rates. TN, NO_3_^−^, and NH_4_^+^ had almost entirely different effects, and their interpretation rates accounted for 8.08%. Significant heavy metals included As, Mn, and Ni, and their influences accounted for 7.16%. We note that high correlations (Table S1) were found between some factors, for example, Res-P and TP, TN and TOC, and Cu and Ni. Therefore, the influences of some environmental factors may be due to the related factors.

## 4. Discussion

### 4.1. The Compositions of Fungal Communities in the Sediment Environment of Shallow Lakes

The humus-rich anoxic environments of shallow lake sediments are suitable for fungi, which play important roles in the transformation of plant residues into fodder for invertebrates and other organisms [25]. This study revealed highly diverse communities of fungi in the BYD Lake sediments, covering most known fungi phyla [26]. Studies have found that Ascomycota accounts for a large percentage in the sediment [27,28,29,30], and this was consistent with that in the BYD Lake. However, the proportions of Chytridiomycota and other fungi, such as Cryptomycota, were much higher in the BYD Lake, especially in summer. Chytrids which produce spores with a motile cilium that helps them to swim and/or crawl [31], are adapted to aquatic habitats [32,33] and prefer saprophytic environments with animal and plant residues [34]. In addition, a large number of chytrids parasitize algae [35]. The high proportion of Chytridiomycota in BYD Lake sediment may be related to the abundance of plant residues, or the bloom of algae caused by eutrophication in summer [36]. Cryptomycota species are commonly found as parasites in aquatic and soil ecosystems, and their hosts are mainly Chytridiomycota [37]. Therefore, the abundance of this phylum may related to the abundance of Chytridiomycota. 

Most zygosporic species are terrestrial fungi [38] and are rare in aquatic habitats. In contrast, Glomeromycota form symbionts of arbuscular mycorrhiza with plants [39], and common reeds (*Phragmites australis*) in BYD Lake were commonly colonized [40]. It was unexpected that few Glomeromycota species were identified in this study. We guess that the primers may not be suitable for PCR amplification of this phylum, for at present there is no better way to identify endophytic fungi, which include most Glomeromycota [41]. Otherwise, those norank fungi (*NG_Fungi*) or unclassified fungi (*UG_Fungi*) that account for large proportions may belong to Glomeromycota, as most genomes of arbuscular mycorrhizal fungi have not been revealed, and the genomes of only a few species have been published in recent years [42,43,44,45]. 

In addition to fungi, a great number of eukaryotes species were identified from the OTUs (accounted for almost half sequence numbers, Table S2). Most of them were flagellates or ciliates, belonging to Choanozoa or Ciliophora. Unexpectedly, only a handful algae species were identified, and most of them belong to Cryptophyta, which consist of single-celled algae with two flagella. These microorganisms have similar physiological structures and closer phylogeny with fungi, as cilium or flagellum are also present in fungi [46]. This may be the reason why these species were present in the PCR products.

### 4.2. The Temporal and Spatial Variations of Fungal Communities

The temporal and spatial dynamics of fungal communities have been revealed in freshwater ecosystems, but researchers have not reached a consensus on whether seasons or habitats have a greater influence [47]. In this study, the results of ANOSIM (Figure S2) indicated a high specificity of fungal communities in different habitats. When samples from the same season were composed and analyzed, there was almost no difference between intragroup and intergroup differences. This indicates that habitat has a higher influence on the compositions of fungal communities. It was contrary to the α diversity indexes that showed highest biodiversity in winter and no significant differences among most sites (Figure 4). This may be related to how the α diversity indexes were calculated. We calculated α diversity indexes using species numbers and proportions of species, so no significant difference among sites indicates that each site has similar species numbers and similar percentages of each species. Meanwhile, the β diversity verified that the compositions of fungal communities differed among sites, especially those in lotus ponds and duck farms. The dominant genera shown in Figure 3 indicates that the most dominant fungi maintained their dominance across seasons. On the other hand, other taxa, which comprised a relatively small part in the number of OTUs but composed a considerable part of the species numbers, resulted in higher α diversity index. 

The effects of habitats on fungal communities were also manifest in their compositions and biomass. Figure 5 shows that the fungal communities of lotus ponds and duck farms were most different. Similarity analysis (Table S4) revealed that lotus ponds had significantly higher percentages of *UG_Fungi*, *NG_Cryptomycota*, and *UG_Dothideomycetes*, while duck farms have more *UG_Trichocomaceae*, *Pseudallescheria*, *Talaromyces,* and *NG_Sordariales*. Some fungi only thrive in habitats with intense human activities. For instance, *Pseudallescheria boydii* is a common human pathogen [48], and in this study we found that this species was only enriched in S6, S7, S8, and S9/S9*, where there were more human activities. There may be more species related to human activities present, but it is hard to judge due to the poor understanding of their taxonomy and ecological functions. Moreover, although the fungal diversity was not significantly lower in fish farms than other habitats, the sequence numbers were the lowest in S4 and S5. This probably correlated with fish cultivation measures that inhibit microorganisms, as we found a good number of OTUs identified as Ichthyosporea (Table S2), which include several common fish parasites [49] in those sites. 

### 4.3. The Factors Affect Sediment Fungal Community Compositions

Globally, more fungal species concentrate in temperate and cold temperate zones [50], indicating that fungi are generally more tolerant to the cold than the heat. Researchers also found that fungi were more active in winter, as they detected most enzymes in winter than other seasons in soil [51]. The results of this study are consistent with this opinion. There are more species in winter and some dominant genera are psychrophilic, for example, *Cladosporium* and *Mrakia* [52]. The psychrophilic fungi were common in winter but rare in summer. However, there were also some fungi adapted to high temperature. For example, *Talaromyces*, which is a mold in the environment and a yeast in the tissues at high temperature [53], was rare in winter or spring but common in summer. The percentages of the parasitic fungi Cryptomycota were much higher in summer, and this may be related to their higher biological activities at higher temperatures. Meanwhile, the influence of temperature on Chytridiomycota was not significant since the percentages of Chytridiomycota did not vary greatly among seasons. 

Researchers have different views about the effects of nutrients on microorganisms, suggesting that phosphorus and nitrogen may have positive or negative effects [28,54], and the types of nutrient exert different degrees of effects on the fungal structure [55]. According to the results of RDA in Figure 6, the arrows standing for NO_3_^−^ and NH_4_^+^ had opposite directions, indicating that they have opposite effects on the fungal communities. In addition, their effects were almost completely different from those of TN, which were mainly composed of organic nitrogen [56]. These may be due to the difference between mycorrhizal fungi and saprophytic fungi in the utilization of different nitrogen sources [57,58,59]. However, there was no evidence that certain fungus species were capable to take up nitrogen in a certain form, partly because most fungal species were poorly identified. In addition, TN and TOC were highly correlated in this study, and it is hard to distinguish which one is the real driving parameter. Studies have verified that soil microorganism communities are affected by active phosphorus [54], as P is one of the most important macronutrients for organisms. Unexpectedly, inert Res-P also showed a significant influence and had the highest interpretation rate among all P forms. This indicates that some fungi in the sediment may be effective phosphate-solubilizers [60].

In most cases, the accumulation of heavy metals causes adverse effects on microorganisms [61], but some microbial communities can develop a functional resistance to the stress of heavy metals [62]. Consequently, fungi has been used for the detoxification of heavy metals [63]. However, in this study, except for Cr, there was no significant difference in heavy metal concentrations between the sediments and the regional background values (Table S3, [64]), and the influence of Cr on fungal communities was not significant. Some studies have illustrated that heavy metals have no influence on microbial community compositions [65]. The RDA results showed that heavy metals, including As, Mn, and Ni, or maybe the high-correlated Cu, have contributed to the interpretation of the influence of environmental factors on fungal community composition, even though the contributions were relatively low. Unfortunately, we did not identify any known tolerant fungal species [63] in this study, so we are not sure how fungi are influenced by heavy metals in BYD Lake sediments. These mechanisms require more investigation.

### 4.4. Deficiencies of the Study

The development of environmental DNA sequencing technology has enabled an increasing number of microorganisms to be discovered, but traditional taxonomy based on morphology is far behind in development, and a large number of new species are yet to be described. At the same time, research on the ecological habits and functions of fungi, as well as other microorganisms, is not thorough enough to understand the role they play in ecosystems. For example, among the fungal species classified in this study, 35 species from 167 were labeled as uncultured, which means that they are currently only found in nature through DNA sequence analysis and could not be obtained through culture experiments. There were also 50 species unclassified. The physiology and ecological habits of these fungi are basically unknown. Exploring their roles in ecosystems will be an important challenge in the future study of lake ecosystem functions.

Limited by personnel and funds, we only sampled for three seasons, so the analyses were not rigorous. In addition, seasonal dynamics of fungal communities are less profound in deep layers than the top [65]; mixing the samples may weaken the dynamics and lead to inaccurate results. In addition, in this study, only the indirect effects of changes in environmental factors caused by human activities on the fungal community were discussed, and we failed to develop a quantitative division of the intensity of human activities. Therefore, it was not enough to evaluate the impact of human activities on the fungal community in the BYD Lake sediments. More scientific experiments still need to be performed.

## 5. Conclusions

High-throughput sequencing technology provides a convenient means for detecting microbial diversity in the environment, but there are still many shortcomings. More than 100 fungal species have been identified in the sediment of BYD Lake, but most of their information is not complete, including some important fungi, such as Glomeromycota. More studies are required to further explore their ecological functions. 

In the sediment of temperate shallow lakes, more fungal species prefer a cold environment, and the number of rare species in winter is significantly higher than other seasons, resulting in higher biodiversity in winter. There was no significant difference in fungal diversity indexes among different habitats, while the composition of the communities was quite different. The impacts of human activities on fungal communities were not as strong as expected. Some human-related fungi only concentrated in habitats with intensive human activities, and fish cultivation measures may lead to a lower fungal biomass in fish farms.

Environmental factors have various effects on fungal communities. In general, the most influential factor, temperature, is negative to fungal diversity, but some fungi, such as Cryptomycota, are dominant at high temperatures. Different forms of N (NO_3_^−^, NH_4_^+^, and organic N) play almost completely different roles in shaping the fungal community. Different forms of P also showed different effects. More research is needed to reveal the effects of heavy metals.

## Figures and Tables

**Table 1:** Characteristic values of environmental factors in sediment of BYD Lake.

Env. Factors	Minimum~Maximum			Average ± Standard Deviation		
	Winter	Spring	Summer	Winter	Spring	Summer
T (°C)	−2.61~−0.24	6.10~7.80	26.40~28.50	−1.77 ± 0.60	7.06 ± 0.49	27.27 ± 0.41
pH	7.66~8.33	6.90~7.93	7.09~7.62	7.93 ± 0.20	7.44 ± 0.22	7.43 ± 0.15
TP (mg/kg)	568~1734	473~3101	496~3653	962.6 ± 296.5	960.5 ± 529.4	1071.9 ± 599.9
Res-P (mg/kg)	144.7~588.8	153.6~449.7	36.7~1134.8	304.63 ± 111.84	185.09 ± 68.24	234.13 ± 212.80
HCl-P (mg/kg)	276.5~914.5	235.7~2109.9	340.6~2175.7	529.25 ± 164.12	610.12 ± 376.78	642.33 ± 362.42
NaOH-P (mg/kg)	48.2~204.4	69.1~299.8	83.0~338.6	111.06 ± 44.49	96.77 ± 48.16	158.46 ± 54.96
BD-P (mg/kg)	1.4~18.8	9.8~144.1	0.2~80.2	11.33 ± 4.58	50.37 ± 32.17	24.44 ± 26.80
NH_4_Cl-P (mg/kg)	0.78~19.74	0.08~25.22	0.02~47.59	5.86 ± 5.37	9.28 ± 8.90	12.45 ± 16.63
TN (g/kg)	0.70~12.40	9.76~13.24	1.37~6.82	3.70 ± 2.44	10.49 ± 3.66	3.75 ± 1.73
NO_3_^−^ (mg/kg)	1.13~8.93	0.98~3.21	0.80~6.77	4.59 ± 1.80	1.64 ± 0.73	2.13 ± 1.62
NH_4_^+^ (mg/kg)	0.84~5.26	2.38~6.25	1.78~12.21	2.12 ± 1.14	4.10 ± 1.03	6.56 ± 3.19
TOC (g/kg)	4.34~132.95	28.22~115.65	12.97~66.83	36.02 ± 26.70	83.30 ± 21.91	36.44 ± 17.63
As (mg/kg)	5.66~16.90	3.48~16.10	4.88~12.88	10.32 ± 2.73	10.06 ± 2.99	9.12 ± 1.81
Cd (mg/kg)	0.07~2.36	0.07~0.71	0.16~0.75	0.49 ± 0.47	0.43 ± 0.19	0.43 ± 0.13
Cr (mg/kg)	48.60~80.10	35.00~248.90	21.00~97.0	61.30 ± 8.84	83.85 ± 53.75	49.93 ± 15.83
Cu (mg/kg)	16.70~57.90	16.00~74.30	20.10~72.10	32.53 ± 11.58	38.06 ± 16.09	41.53 ± 15.76
Pb (mg/kg)	16.20~44.70	11.20~26.20	14.60~34.40	25.68 ± 6.75	19.60 ± 3.85	22.37 ± 4.86
Zn (mg/kg)	54.60~244.00	54.20~332.80	67.20~249.00	112.11 ± 49.15	115.99 ± 54.73	119.80 ± 41.48
Co (mg/kg)	7.39~13.70	3.50~12.02	7.83~13.22	11.12 ± 2.19	8.89 ± 1.94	10.10 ± 1.48
Mn (mg/kg)	448~796	403~915	420~751	600.46 ± 90.26	602.28 ± 146.86	622.61 ± 95.35
Ni (mg/kg)	20.00~30.30	19.20~131.60	23.60~83.50	30.12 ± 9.62	45.25 ± 29.48	40.70 ± 17.28
Fe (g/kg)	23.26~33.24	10.78~35.74	24.95~37.68	29.24 ± 4.71	26.12 ± 5.77	30.32 ± 3.60

**Table 2:** Interpretation rates (R^2^) of significant environmental factors on variations of fungal community compositions.

Factors	T	Res-P	NaOH-P	BD-P	NH_4_Cl-P	TN	NO_3_^−^	NH_4_^+^	As	Mn	Ni
**R^2^**	22.47%	3.98%	1.71%	2.72%	3.25%	4.87%	1.70%	1.51%	1.91%	2.33%	2.60%
***p***	0.001	0.001	0.018	0.002	0.001	0.001	0.018	0.049	0.003	0.006	0.001

## Data Availability

All relevant data are within the manuscript and the Supplementary Materials.

## Supplementary Materials

The following supporting information can be downloaded at: https://www.mdpi.com/article/10.3390/microorganisms12112127/s1, Figure S1. Contents of sediment physical and chemical properties among all habitats in three seasons. Error bars represented the standard deviations. Figure S2. ANOSIM results of fungal communities in different sites and seasons: (a) sites, (b) seasons. Table S1. Pearson relationships of the environmental factors of Baiyangdian Lake sediments. Table S2. Sequence numbers of fungi at phylum level in BYD Lake sediments. Table S3. Background value of the concentrations of heavy metals in soil of Hebei Province. Table S4. Key genera contributing to similarity of fungal composition between lotus ponds and duck farms.
