# Supplementary material for: Seasonal Variations of Sediment Fungal Community of a Shallow Lake in North China
Source: Microorganisms. 2024 Oct 24;12(11):2127. doi: 10.3390/microorganisms12112127 (PMC11596378; doi:10.3390/microorganisms12112127)
Supplement: Supplementary file 1 [file microorganisms-12-02127-s001.zip › microorganisms-3203051-supplementary.pdf]

Supporting Information

Seasonal variations of sediment fungal community of a shallow lake in north China

The file includes:

Six pages

Table S1 to S4

Figure S1 to S2

Table S1. Pearson relationships of the environmental factors of Baiyangdian Lake sediments

	T	TP	Res-P	HCl-P	NaOH-P	BD-P	NH_4Cl-P	TN	NO_3^-	NH_4^+	pH	TOC	As	Cd	Cr	Cu	Pb	Zn	Co	Mn	Ni	Fe
T	1																					
TP	0.10	1																				
Res-P	-0.13	**0.80****	1																			
HCl-P	0.13	**0.98****	0.67*	1																		
NaOH-P	0.41**	0.76**	0.60*	0.70**	1																	
BD-P	0.07	0.36**	-0.15	0.49**	0.06	1																
NH_4Cl-P	0.23*	0.03	-0.28*	0.13	-0.15	0.48**	1															
TN	-0.16	-0.10	-0.24*	-0.04	-0.24*	0.42**	0.02	1														
NO_3^-	-0.41**	0.07	0.14	0.05	0.01	-0.11	0.11	-0.30**	1													
NH_4^+	0.66**	-0.06	-0.28*	-0.04	0.35**	0.18	0.18	0.05	-0.32**	1												

pH	-0.59**	-0.36**	0.02	-0.44**	-0.40**	-0.54**	-0.12	-0.38**	0.38**	-0.49	1												
TOC	-0.15	-0.05	-0.21	0.01	-0.13	0.41**	-0.05	**0.96****	-0.25	0.07	-0.39*	1											
As	-0.20	-0.11	-0.05	-0.13	-0.05	0.03	-0.19	0.18	0.10	0.01	0.02	0.27	1										
Cd	-0.07	0.18	0.12	0.17	0.30*	0.02	-0.09	0.22*	0.18	0.04	-0.13	0.37**	0.58	1									
Cr	-0.21	0.37**	0.22*	0.39**	0.07	0.46**	-0.05	0.17	-0.03	-0.17	-0.27*	0.20	0.19	0.06	1								
Cu	0.23*	0.35**	0.17	0.33**	0.51**	0.37**	-0.13	0.28*	-0.07	0.45**	-0.56**	0.37**	0.36**	0.43**	0.40**	1							
Pb	-0.15	0.22	0.27*	0.15	0.34**	-0.06	0.14	-0.05	0.40**	0.03	0.09	0.03	0.49**	0.69**	-0.05	0.34**	1						
Zn	0.06	0.69**	0.49**	0.68**	0.61**	0.38**	0.03	0.05	0.11	0.07	-0.49**	0.11	0.21	0.38**	0.46**	0.64**	0.43**	1					
Co	-0.10	0.14	0.18	0.09	0.16	0.05	0.24*	-0.08	0.44**	-0.02	0.10	-0.12	0.07	0.13	-0.05	0.25*	0.62**	0.30**	1				
Mn	0.09	-0.14	-0.09	-0.17	0.04	0.07	-0.15	0.13	-0.01	0.23	-0.13	0.19	0.45**	0.12	0.23*	0.54**	0.19	0.11	0.27*	1			
Ni	0.15	0.10	-0.05	0.11	0.19	0.41**	-0.15	0.39**	-0.18	0.35**	-0.43**	0.44**	0.30**	0.17	0.53**	0.83**	-0.01	0.29**	0.05	0.63*	1		
Fe	0.17	0.12	0.06	0.11	0.16	0.10	0.33**	-0.07	0.23*	0.15	-0.06	-0.14	-0.04	0.11	-0.15	0.17	0.48**	0.22*	**0.88****	0.19	-0.05	1	

Note: * represents $p < 0.05$; and ** $p < 0.01$

Table S2. Sequences numbers of fungi at phylum level in BYD Lake sediments

Sites / Taxa	S1	S2	S3	S4	S5	S6	S7	S8	S9/S9*
**Fungi**	**201925**	**224788**	**151933**	**106791**	**122722**	**239689**	**166587**	**200683**	**199820**
Ascomycota	82235	65671	30534	42168	29411	96212	41567	68696	121616
Dothideomycetes	24492	32527	10452	10939	6922	10711	8024	15933	11722
Eurotiomycetes	14794	7078	4919	6396	5948	24922	9232	13677	37707
Saccharomycetes	4353	2488	1684	1425	1781	17529	1974	5442	9836
Basidiomycota	11541	5379	8308	8070	3964	12139	8179	10033	11041
Blastocladiomycota	1497	3856	2259	2052	3316	3475	3447	5335	453
Chytridiomycota	34713	68082	56137	31569	43536	60155	46171	59507	31059

Cryptomycota	30637	28656	12358	4809	13280	17111	14986	13298	6021
Entomophthoromycota	0	0	0	0	0	4	0	0	0
Glomeromycota	7	0	0	188	0	7	0	69	0
Kickxellomycota	62	1227	104	362	501	553	435	640	452
Neocallimastigomycota	1263	195	310	242	872	1609	702	667	150
Zoopagomycota	383	0	0	530	0	0	70	0	0
norank Fungi	12158	15168	8375	1579	2171	6864	6603	4827	2248
unclassified Fungi	27429	36554	33548	15222	25671	41560	44427	37611	26780
**Protozoa**	**101978**	**92829**	**115550**	**173268**	**157048**	**60497**	**118559**	**109573**	**93944**
Choanozoa	26703	41080	39345	74804	52268	32690	30756	27873	12783
Ichthyosporea	1514	3147	1472	50251	26331	2487	2159	1464	1449
Ciliophora	59669	37326	64227	81340	87554	17238	81582	66266	20686
other protozoa	15606	14423	11978	17124	17226	10569	6221	15434	60475
**Algae**	**17700**	**18877**	**58256**	**14964**	**22516**	**2374**	**3746**	**10355**	**8716**
Cryptista	15261	18091	57368	10894	20279	1812	3214	9140	8228
other algae	2439	786	888	4070	2237	562	532	1215	488
**other Eukaryotes**	**33085**	**13749**	**16146**	**25858**	**24029**	**19957**	**17021**	**13559**	**17613**
**Total**	**354688**	**350243**	**341885**	**320881**	**326315**	**322517**	**305913**	**334170**	**320093**

Table S3. Background value of the concentrations of heavy metals in soil of Hebei Province
The values derived from the summary of China Environmental Monitoring Center (1990).

**Heavy Metals**	**Average Concentrations of background soil ± Standard Deviation(mg/kg)**
**As**	13.6 ± 5.11

**Cd**	0.094 ± 0.0792
**Cr**	68.3 ± 22.35
**Cu**	21.8 ± 6.22
**Pb**	21.5 ± 6.88
**Zn**	78.4 ± 38.19
**Co**	12.4 ± 3.91
**Mn**	608 ± 137.2
**Ni**	30.8 ± 11.18
**Fe**	2820 ± 690

Table S4. Key genera contributing to similarity of fungal composition between lotus ponds and duck farms

**Genus**	**Contribution percentage**	**Average relative abundance**	
		lotus ponds	duck farms
unclassified_genus_Fungi	15.02%	10.3%	8.1%
norank_genus_Chytridiomycota	12.05%	5.6%	5.5%
unclassified_genus_Trichocomaceae	9.74%	1.5%	7.7%
norank_genus_Cryptomycota	9.58%	2.6%	1.2%
Pseudallescheria	4.39%	0.5%	7.3%
norank_genus_Sordariomycetes	4.16%	0.7%	2.1%
unclassified_genus_Dothideomycetes	4.00%	5.1%	1.1%
Talaromyces	3.67%	0.1%	1.7%
Cladosporium	3.36%	0.4%	1.5%
norank_genus_Sordariales	3.35%	0.4%	.4.4%

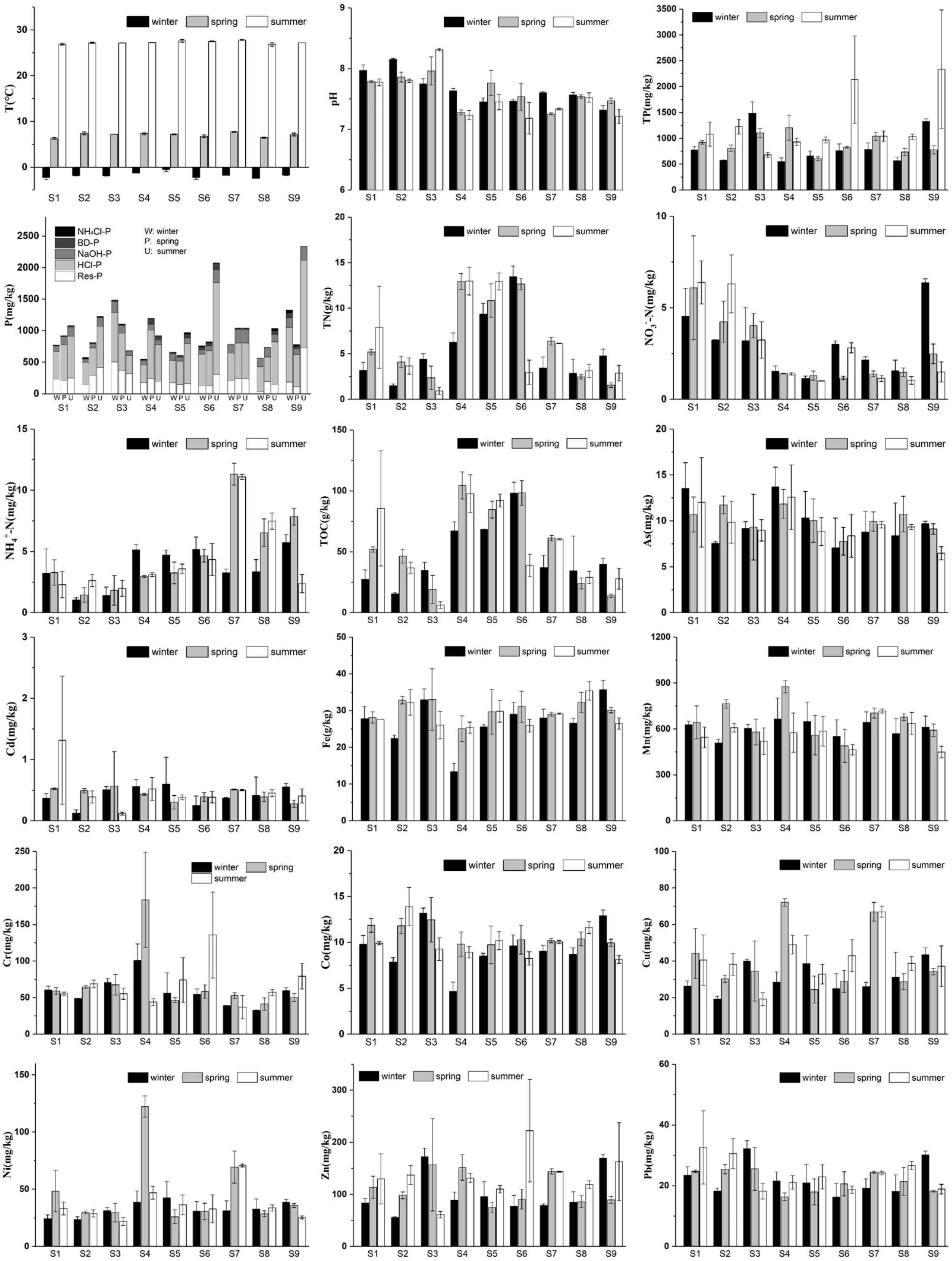

Figure S1. Contents of sediment physical and chemical properties among all habitats in three seasons. Error bars represented the standard deviations.

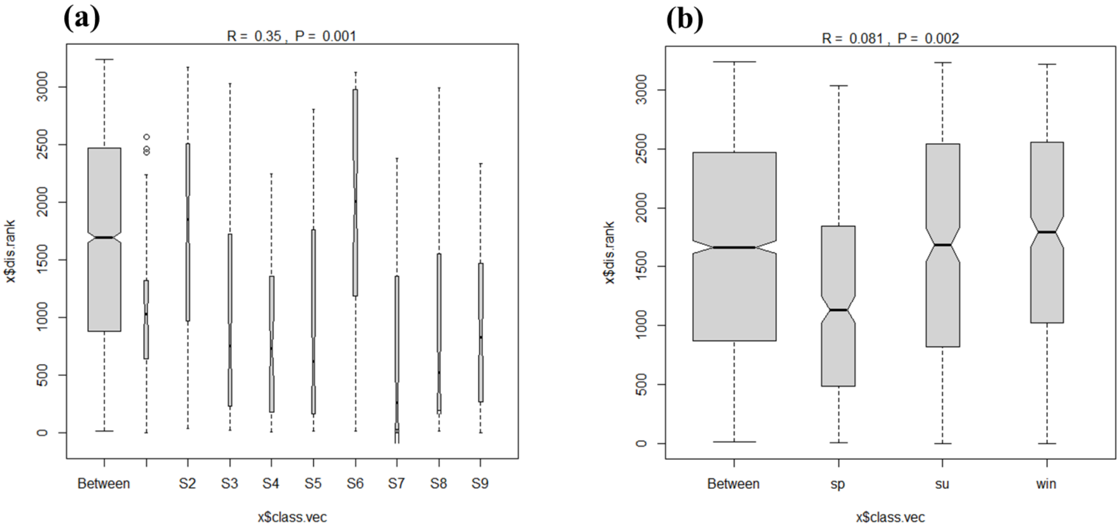

Figure S2. ANOSIM results of fungal communities in different sites and seasons. (a) sites; (b) seasons

References

China Environmental Monitoring Center. *Background Value of Soil Elements in China*; China Environmental Science Press: Beijing, China, 1990.
